# The Association of Maternal Emotional Status With Child Over-Use of Electronic Devices During the COVID-19 Pandemic

**DOI:** 10.3389/fped.2021.760996

**Published:** 2021-12-06

**Authors:** Xiangrong Guo, Yulai Zhou, Jian Xu, Yuelai Hu, Zhiwei Liu

**Affiliations:** ^1^The International Peace Maternity and Child Health Hospital, Shanghai Jiao Tong University School of Medicine, Shanghai, China; ^2^Shanghai Key Laboratory of Embryo Original Diseases, Shanghai Jiao Tong University School of Medicine, Shanghai, China; ^3^Ministry of Education (MOE)-Shanghai Key Lab of Children's Environmental Health, Xinhua Hospital, Shanghai Jiao Tong University School of Medicine, Shanghai, China; ^4^Shanghai Pinghe Bilingual School, Shanghai, China

**Keywords:** COVID-19 pandemic, maternal depression, maternal anxiety, child electronic devices over-use, media, family environment

## Abstract

The quarantine during the COVID-19 pandemic may generate high levels of maternal depression/anxiety, and maternal emotional status may affect child behavioral development. Online education during the pandemic may induce child over-use of electronic-devices. However, child electronic-device over-use (especially among children under 12 who are immature in physical and mental development) during the pandemic has not attracted sufficient attention, and the association of child over-use with maternal emotional status remains unknown. Therefore, this study aims to assess the characteristics of child electronic-device over-use and the association between maternal emotional status and child over-use among 1,300 children from nurseries (<3 years), kindergartens (3–6 years), and primary schools (6–12 years) in Shanghai and Wuhan during COVID-19. Mothers completed an online questionnaire (including the Self-Rating-Depression/Anxiety-Scales and Family-Environment-Scale). The use of electronic devices (mobile-phones, iPads, computers, and televisions) and online courses taken by the children were investigated. Associations of maternal emotional status with electronic-device-use by child age were analyzed. The proportions of children in nurseries, kindergartens and primary schools were 8.5, 44.5, and 47.0%, their percentages following online-courses were 24.5, 48.4, and 99.0%, and their rates of electronic-device over-use were 34.2, 62.2, and 93.4%, respectively. Significant associations were observed between higher maternal anxiety/depression levels and higher risks of mobile-phone/iPad over-use among preschoolers and primary-school students. Lower family intimacy and higher conflict levels were associated with higher maternal depression/anxiety levels and higher risks of electronic-device over-use. Our findings suggested that over-use of electronic-devices among children under 12 was common during COVID-19, especially among children ≥6 years, and online-teaching may exacerbate over-use. Maternal anxiety/depression levels were associated with over-use of portable internet-devices (mobile-phone/iPad), especially among preschoolers and school-aged students, and family environment may mediate the association. These findings may contribute to a better understanding of factors leading to over-use of electronic-device and developing strategies to decrease over-use during COVID-19.

## Introduction

The Coronavirus Disease 2019 (COVID-19) has spread globally and affected millions of people (1, 2). So far, there have been more than 234 million cases and 4 million deaths (3). To control the spread of the COVID-19, strict domestic quarantine was implemented, schools were closed with online courses developed for children to study at home instead (4, 5). However, in these cases, it may cause emotional problems of mothers and the risk of child over-use of electronic products.

Domestic quarantine may affect people's emotional well-being and increase the risks of post-traumatic stress symptoms, confusion, and anger (6, 7). A recent study during the COVID-19 pandemic demonstrated that women reported higher levels of post-traumatic stress symptoms in the domains of re-experiencing, negative alterations in mood or cognition, and hyper-arousal than men (8). Compared with fathers, mothers may engage in more activities with children (9, 10). Another study conducted during the COVID-19 pandemic has shown that mothers' employment was more affected relative to fathers', and mothers of young children have reduced their work hours significantly more than fathers (especially true for mothers of children under 12 years old) (11). Therefore, mothers may experience high levels of depression/anxiety during the COVID-19 pandemic probably because of maternal worries about the risks of their children/other family members getting COVID-19, a great child-care/homeschooling burden (mothers may undertake more child health-care/homeschooling tasks than usual because children stay at home all day due to school closures), and the risk of unemployment.

With advances in technology and improved living standards, an increasing number of children are exposed to electronic devices such as televisions (TVs), mobile phones, iPads, and computers. Children aged 0–12 years are at an immature but important developmental stage of physical and mental development. Excessive use of electronic devices may cause a range of problems, such as somatization, myopia, obesity, sleep disorders, and impaired social skills (12–14). About the definition of child over-use of electronic devices, according to the latest guidelines from the American Academy of Pediatrics, children under 2 years of age shall strictly restrict the use of electronic devices; children of 2–5 years old cannot watch electronic programs for more than an hour per day; while children aged 5–12 years cannot use electronic devices for more than 1.5 h per day (15). In China, during the COVID-19 pandemic, almost all the school-age children and some younger children took online classes with the assistance of video conferencing apps such as Ding Talk and Tencent Meeting. Online courses extend the use-time of electronic-devices and may have an unfavorable impact on child emotional and physical health (16). Therefore, in such a new situation, the characteristics of child electronic-device use may be different from those before the pandemic which are new challenges in the control of child media use, and warrant further investigations.

Thus, mothers may suffer from high levels of depression/anxiety during the COVID-19 pandemic. As maternal anxiety or depressive symptoms may affect the way mothers raise their children (11), maternal emotion may exert a profound influence on child emotional and behavioral development. Understanding the characteristics of child over-use of electronic devices and the association between maternal emotional status and child over-use may help to develop strategies to promote positive mother-child interaction and appropriate use of electronic devices in children, which could be critical to the child's healthy growth and development. Therefore, we hypothesized that child electronic-device over-use was associated with maternal emotional status during the COVID-19 pandemic, and the association might be different by child age.

## Materials and Methods

### Study Design and Recruitment

During March to April 2020 (the COVID-19 pandemic period), we recruited a convenience sample from 15 schools (one nurseries/four kindergartens/10 primary schools) in Shanghai and six schools (one nurseries/four kindergartens/one primary school) in Wuhan, and conducted an online survey (17). The recruited schools in each city had diversity in geographic location and socio-economic status. Due to the policy of home isolation, the questionnaire was distributed to the mothers of the participating children by the teachers in charge of the classes *via* online class groups. Before distributing the questionnaire, a brief introduction and guidance on how to fill out the questionnaire were provided to the mothers by WeChat class groups. All students involved in the routines in the classrooms in the study schools before the pandemic were invited to participate in this study. Students were excluded if the mothers had mental illnesses/disorders during the study. Ultimately, a total of 1,315 questionnaires were collected, and 1,300 questionnaires were included in the final statistical analysis because 15 questionnaires were excluded due to double submission or logic errors detected in data cleaning.

The Research Ethics Committee Board of the International Peace Maternity and Child Health Hospital approved this study. Informed consents were obtained from all participants.

### Measurement

#### Assessment of Maternal Emotional Status

The Chinese versions of the Self-rating Depression Scale (SDS) (18) and the Self-rating Anxiety Scale (SAS) (19) were the most widely used measures in China for screening depressive and anxiety symptoms, respectively, and were used to measure maternal depression and anxiety levels in this study. The SDS and SAS both contained 20 items. Each item was rated on a score from 1 (none, or a little of the time) to 4 (most, or all of the time), and a raw total score was generated by summing the item scores. Higher SDS and SAS index scores (raw total scores^*^1.25) indicated higher depression and anxiety levels, respectively. Based on the results of Chinese norms, the presence of depressive symptoms was defined as a SDS index score ≥53, and the presence of anxiety symptoms was defined as a SAS index score ≥50. The SDS and SAS Chinese versions were reliable tools, and had good reliability (these two scales had good internal consistency, with Cronbach's alphas of 0.86 for the SDS Chinese version and 0.93 for the SAS Chinese version) (20, 21). These two scales also had good validity in Chinese population through the analyses of content validity, criterion-related validity and structural validity (21–23).

#### Assessment of Family Environment

Interaction/relationship characteristics among family members were evaluated by the Chinese version of the Family Environment Scale (FES-CV) (24). The scale was originally developed by Moos et al. in 1974 (25) and translated into the Chinese version by Wang et al. (26). The FES-CV evaluated the family environment with 90 items categorized into 10 subscales. Among the 10 subscales, the cohesion subscale evaluated the level of commitment, help or support among family members, and the contradiction subscale evaluated the degree to which family members openly expressed anger and disagreements. Higher cohesion/contradiction subscale scores indicated higher intimacy/conflict levels. The cohesion and contradiction subscales of the FES-CV had acceptable reliability and validity (for cohesion: Cronbach α = 0.75; for contradiction: Cronbach α = 0.67) (19), and both worked well in assessing different kinds of families and measuring family relationships/environment in China (27, 28).

#### Assessment of Child Electronic-Device Use

In this study, electronic devices children used included the TV, mobile phone, iPad, and computer. The response options included: “hardly used,” “<0.5 h per day,” “0.5–1 h per day,” “1–2 h per day,” “2–5 h per day,” and “more than 5 h per day.” Because previous studies showed that internet addiction was diagnosed mainly based on excessive use of electronic devices with internet access, and among internet devices, portable internet devices were more likely to induce internet addiction compared with heavier or larger internet devices (such as computers) (29, 30). Therefore, the new variable of “portable internet devices” was created, and the daily usage hours of portable internet devices were calculated by averaging the daily usage hours of mobile phones and iPads.

Based on the latest recommendations of the American Academy of Pediatrics about the daily hours spent on electronic use for children of different ages (15), we divided child electronic-device use into three categories: “appropriate use,” “probably excessive use,” and “excessive use.” “Appropriate use” was defined as the total time children spent on electronic devices per day less than the recommended time, while “excessive use” as the total time more than the recommendation. Since daily use of each electronic device was categorized from “hardly use” to “>5 h/day,” “may be excessive” was applied to the cases when the total time was probably more than the recommendation. All the assessments were conducted according to the criteria corresponding to different age stages.

### Confounders

Confounders belonging to child factors included child gender, age, ethnicity, gestational week at birth, single child or not, parity, and taking online courses (not taking, taking online courses provided by schools, taking online courses provided by private educational companies).

Confounders belonging to maternal and family factors included maternal education, age, marital status, maternal attention/awareness/confidence of healthy parenting during COVID-19 pandemic (a little, very much), residency (urban, sub-urban, and rural), family structure, main child healthcare provider, and if their family members or close relatives were infected with COVID-19 (no case, suspected case, confirmed mild case, confirmed severe case).

### Statistical Analysis

The demographic characteristics of the participants were presented as means and standard deviations (continuous variables) or frequency and percentages (categorical variables). One-way ANOVA (Analysis of variance) and Student-Newman-Keuls *post-hoc* tests were used to compare the differences in maternal depression and anxiety levels among different categories. Infants/toddlers in nurseries, preschoolers in kindergartens and school-aged children in primary schools automatically formed three age groups (<3, 3–6, and 6–12 years old). The Chi-square test was used to compare child over-use of electronic devices among these three age groups. The risk factors of maternal depression and anxiety and child excessive use of electronic devices were analyzed by univariate logistic regression analyses. Multi-variable logistic regression models were applied to explore the adjusted associations between maternal depression/anxiety levels, family intimacy/conflict levels and child electronic over-use. All the statistical analyses were conducted using version 9.4 SAS software (SAS Institute; Cary, NC, USA) and the graphs were generated through Origin Pro 2020b (Learning Edition, Version 9.7.5.184). The significance level was set at two-tailed *P* < 0.05.

## Results

### The Levels and Risk Factors of Maternal Depression and Anxiety During the COVID-19 Pandemic

**Figure 1:**
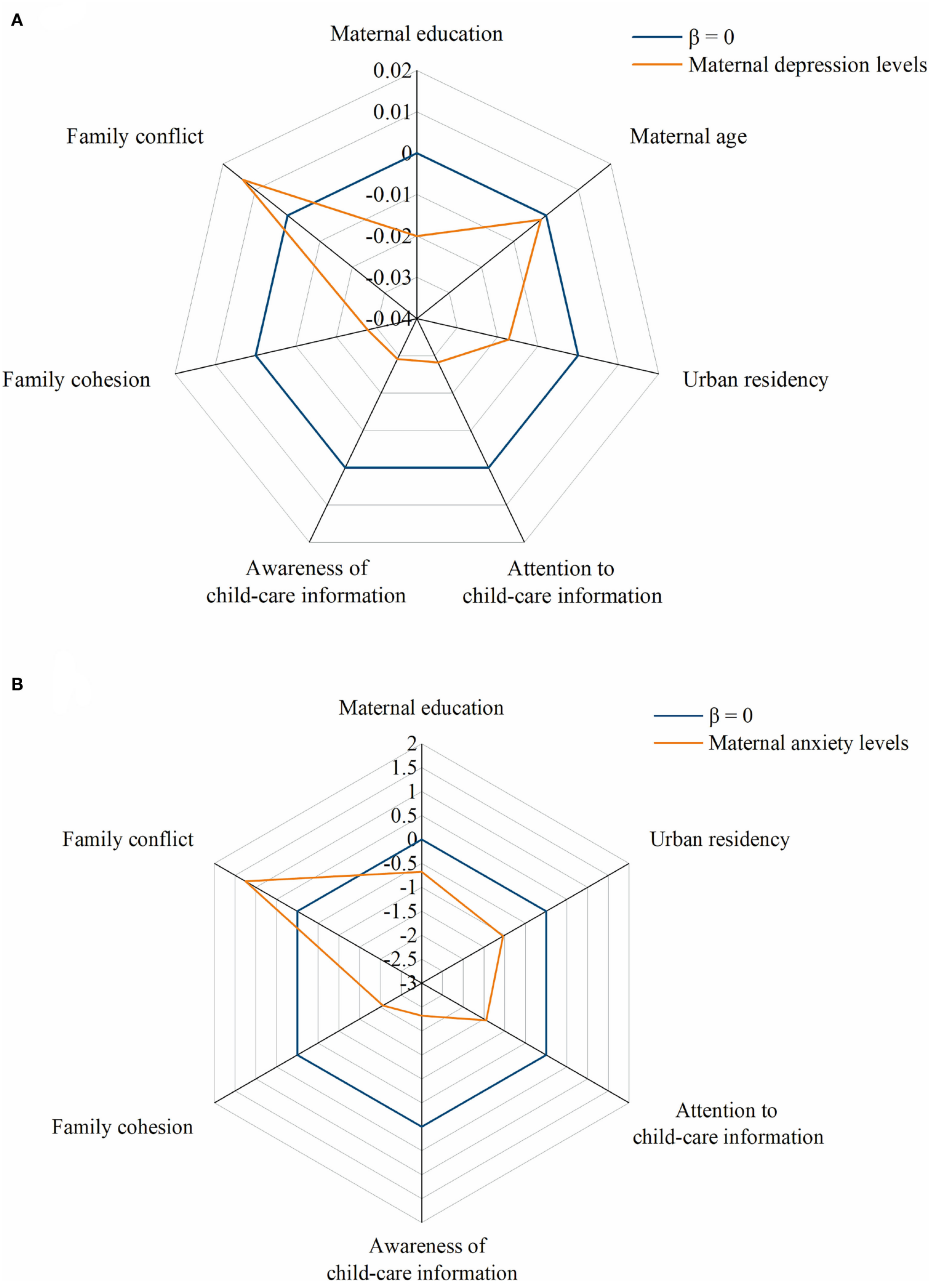
Risk factors for maternal depression and anxiety. Maternal depression **(A)** and anxiety **(B)** levels were both negatively associated with maternal education level, maternal age, home urban residence, attention, and awareness to child-care information during the COVID-19 pandemic and family cohesion levels, and positively related to family conflict levels. Maternal depression levels were also negatively associated with maternal age **(A)**.

The characteristics of the study population were shown in Table 1 and Figure 1. Among the 1,300 children, 8.5% were in nursery schools (<3 years old), and children in kindergartens (3–6 years old) and elementary schools (6–12 years old) accounted for 44.5 and 47.0%, respectively. More than half of the children (52.6%) were the only child of their families.

**Table 1:** Basic characteristics of the study mother-child pairs (*N* = 1,300).

**Variables**		***N* (%) or (mean ± SD)**	**Maternal depression (mean ± SD)**	*P* ^*^	**Maternal anxiety** **(mean ± SD)**	*P* ^*^
Child gender	Male	694 (53.4%)	0.39 ± 0.09	0.330	34.84 ± 7.25	0.350
	Female	606 (46.6%)	0.39 ± 0.09		34.46 ± 7.73	
Child age	<3 yrs	111 (8.5%)	0.38 ± 0.08^a^	0.027	33.02 ± 6.82^a^	0.053
	3–6 yrs	579 (44.5%)	0.40 ± 0.09^b^		34.84 ± 7.42^b^	
	6–12 yrs	610 (47.0%)	0.39 ± 0.10^a,b^		34.80 ± 7.62^b^	
Child ethnicity	Han	1,256 (96.6%)	0.39 ± 0.09	0.540	34.65 ± 7.49	0.824
	Minor ethnicity	44 (3.4%)	0.40 ± 0.10		34.91 ± 7.00	
Child parity	Only child	684 (52.6%)	0.39 ± 0.09^a^	0.633	34.39 ± 7.17^a^	0.306
	Not only child, First-born	297(22.9%)	0.40 ± 0.10^a^		35.16 ± 7.41^a^	
	Not first-born	319 (24.5%)	0.39 ± 0.10^a^		34.79 ± 8.16^a^	
Maternal education	≤Junior High school	42 (3.2%)	0.44 ± 0.11^a^	<0.001	35.74 ± 7.59^a^	0.095
	High school	92 (7.1%)	0.43 ± 0.10^a^		36.01 ± 7.21^a^	
	College	755 (58.1%)	0.39 ± 0.10^b^		34.75 ± 7.19^a^	
	Post-graduate	411 (31.6%)	0.38 ± 0.09^b^		34.09 ± 7.20^a^	
Maternal age		37.0 ± 5.0	0.39 ± 0.09	0.002	34.66 ± 7.48	0.156
Family structure	Nuclear family	768 (59.1%)	0.39 ± 0.09^a^	0.309	34.35 ± 7.52^a^	0.204
	three generation family	499 (3.4%)	0.40 ± 0.09^a^		34.99 ± 7.24^a^	
	Separated parents	11 (0.8%)	0.40 ± 0.11^a^		36.36 ± 8.45^a^	
	Single parents	18 (1.4%)	0.40 ± 0.10^a^		37.72 ± 10.43^a^	
	Reconstituted family	4 (0.3%)	0.48 ± 0.17^a^		35.75 ± 9.60^a^	
Residency	Urban	1,040 (80.0%)	0.39 ± 0.09^a^	0.009	34.49 ± 7.43^a^	0.013
	Sub-urban	244 (18.8%)	0.40 ± 0.10^a^		35.06 ± 7.52^a^	
	Rural area	16 (1.2%)	0.45 ± 0.10^b^		39.75 ± 8.20^b^	
Attention to child- care information	A little	339 (26.1%)	0.41 ± 0.10	<0.001	35.60 ± 7.57	0.007
	Very much	961 (73.9%)	0.39 ± 0.09		34.33 ± 7.42	
Awareness of child-care (*N* = 1,297)	A little	382 (29.5%)	0.42 ± 0.10	<0.001	36.30 ± 7.73	<0.001
	Very much	915 (70.5%)	0.38 ± 0.09		33.95 ± 7.18	
COVID-19 infection among close relatives	No	1,271 (97.3%)	0.39 ± 0.09^a^	0.731	34.64 ± 7.48^a^	0.467
	Suspected but excluded	11 (0.8%)	0.40 ± 0.09^a^		34.09 ± 3.70^a^	
	Confirmed mild case	19 (1.4%)	0.37 ± 0.12^a^		34.37 ± 7.85^a^	
	Confirmed severe case	6 (0.5%)	0.41 ± 0.13^a^		39.50 ± 6.66^a^	
Online course (*N* = 1,298)	Not taking	388 (25.5%)	0.40 ± 0.10^a^	0.135	34.57 ± 7.30^a^	0.878
	Taking school courses	654 (43.0%)	0.39 ± 0.09^a^		34.77 ± 7.65^a^	
	Taking courses from private educational companies	480 (31.5%)	0.39 ± 0.09^a^		34.78 ± 7.28^a^	
Family environment	Cohesion subscale	8.26 ± 1.27	0.39 ± 0.09	<0.001	34.66 ± 7.48	<0.001
	Contradiction subscale	1.93 ± 1.59	0.39 ± 0.09	<0.001	34.66 ± 7.48	<0.001

*yrs, years; wks, weeks*.

* *Analyzed by One-way ANOVA (Analysis of variance)*.

a,b *Analyzed by the Student-Newman-Keuls post-hoc test. Different superscript letters between the two comparison subgroups indicated statistically significant differences between the two subgroups (P < 0.05), while same superscript letters indicated no significant differences (P > 0.05)*.

The average SDS and SAS index scores of the study mothers were 39.25 ± 9.43 and 34.66 ± 7.48, respectively. 15.7 and 4.1% of the study mothers had positive SDS and SAS index scores. The maternal depression level was adversely associated with maternal age and educational level (*P* < 0.05). The average depression level of the mothers of the kindergarten children was higher than that of the mothers of children in nurseries or elementary schools (*P* = 0.027), while the anxiety level of the mothers of the nursery children was slightly lower than that of the mothers of kindergarteners or elementary-school children (*P* = 0.053). The study mothers who lived in urban areas were less likely to be depressed or anxious in comparison with those who lived in the sub-urban or rural areas (*P* < 0.05). Moreover, mothers who learned less knowledge on how to keep children healthy during COVID-19 pandemic were associated with higher maternal anxiety or depression levels (*P* < 0.05).

The cohesion levels were the highest among infant/toddler families, while the contradiction levels were the highest among school-age-child families. Higher maternal anxiety and depression levels were associated with lower intimacy and higher conflict levels (*P* < 0.001) both in unadjusted and adjusted (adjusting for child age, child gender, maternal age, maternal education, family structure, and home residency) models (Supplementary Figure 1).

### The Levels and Risk Factors of Child Over-Use of Electronic Devices

**Figure 2:**
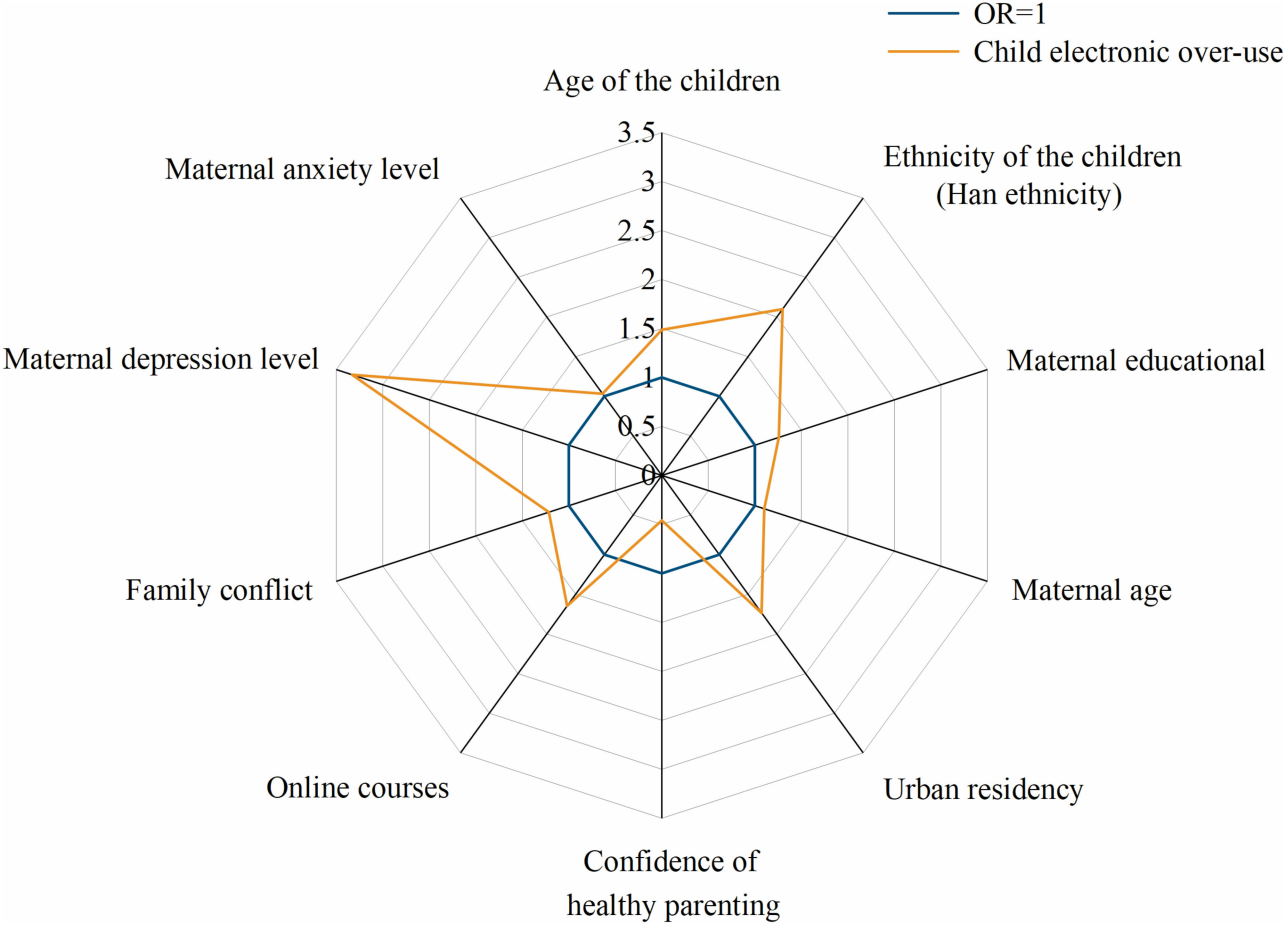
Risk factors for child over-use of electronic devices. Child over-use of electronic devices was positively associated with child age, ethnicity (Han ethnicity), maternal education, maternal age, home urban residency, taking online courses, family conflict levels, maternal depression, and anxiety levels. Child over-use was negatively associated with maternal confidence of healthy parenting during the pandemic.

Among the study children, the percentages of hardly using the mobile phone, iPad and computer were 52.3, 30.9, and 68.8%, indicating that portable internet devices (mobile phone and iPad) were more used than non-portable devices (computer). The percentages of excessive-use or probable excessive-use of electronic devices for children in nurseries, kindergartens and primary schools were 34.2, 62.2, and 93.4%, indicating that older kids might be more prone to spend an excessive amount of time on electronic devices. The risk factors for child excessive use of electronic devices included maternal higher depression or anxiety levels (*P* < 0.05), older children, higher maternal education levels, home urban residency (vs. suburbs and rural areas), ethnicity minorities, lower family intimacy and higher family conflict levels (all *P* < 0.05). Maternal lower confidence in child-care during the pandemic was associated with child excessive electronic use (*P* < 0.001) (Table 2 and Figure 2).

**Table 2:** Child over-use of electronic devices during the COVID-19 pandemic [mean ± SD or *N* (%)].

**Variables**		**Appropriate use (*N* = 332)**	**Maybe excessive use (*N* = 278)**	**Excessive use** **(*N* = 690)**	**Chi-square or *F*-values**	***P-*values**
Child gender	Male	177 (25.5%)	141 (20.3%)	376 (54.2%)	0.27	0.603
	Female	155 (25.6%)	137 (22.6%)	314 (51.8%)		
Child age (yrs)	<3 yrs	73 (65.8%)	25 (22.5%)	13 (11.7%)	341.63	<0.001
	3–6 yrs	219 (37.8%)	159 (27.5%)	201 (34.7%)		
	6–12 yrs	40 (6.6%)	94 (15.4%)	476 (78.0%)		
Child ethnicity	Han	325 (25.9%)	272 (21.6%)	659 (52.5%)	4.67	0.031
	Minor ethnicity	7 (15.9%)	6 (13.6%)	31 (70.5%)		
Child parity	Only child	174 (25.5%)	150 (21.9%)	360 (52.6%)	19.44	<0.001
	Not only child, First-born	55 (18.5%)	58 (19.5%)	184 (62.0%)		
	Not first-born	103 (32.3%)	70 (21.9%)	146 (45.8%)		
Maternal education	≤Junior High school	18 (42.8%)	12 (28.6%)	12 (28.6%)	9.54	0.002
	High school	32 (34.8%)	19 (20.6%)	41 (44.6%)		
	College	182 (24.1%)	163 (21.6%)	410 (54.3%)		
	Post-graduate	100 (24.3%)	84 (20.5%)	227 (55.2%)		
Maternal age (yrs)		35.3 ± 4.8	36.5 ± 4.6	38.1 ± 5.1	75.07	<0.001
Family structure	Nuclear family	202 (26.3%)	173 (22.5%)	393 (51.2%)	3.85	0.427
	Three generation family	124 (24.9%)	99 (19.8%)	276 (55.3%)		
	Separated parents	1 (9.1%)	3 (27.3%)	7 (63.6%)		
	Single parents	5 (27.8%)	2 (11.1%)	11 (61.1%)		
	Reconstituted family	0 (0.0%)	1 (25.0%)	3 (75.0%)		
Residency	Urban	242 (23.3%)	211 (20.3%)	587 (56.4%)	23.23	<0.001
	Sub-urban	85 (34.8%)	62 (25.4%)	97 (39.8%)		
	Rural area	5 (31.3%)	5 (31.3%)	6 (37.44%)		
Attention to child care information	A little	80 (23.6%)	79 (23.3%)	180 (53.1%)	0.25	0.619
	Very much	252 (26.2%)	199 (20.7%)	510 (53.1%)		
Awareness of child-care (*N* = 1,297)	A little	86 (22.5%)	84 (22.0%)	212 (55.5%)	2.15	0.143
	Very much	244 (26.7%)	194 (21.2%)	477 (52.1%)		
Confidence of healthy parenting	A little	19 (12.9%)	27 (18.4%)	101 (68.7%)	18.60	<0.001
	High	313 (27.1%)	251 (21.8%)	589 (51.1%)		
COVID-19 infection among close relatives	No	327 (25.8%)	271 (21.3%)	671 (52.9%)	1.47	0.689
	Suspected but ruled out	1 (12.5%)	2 (25.0%)	5 (62.5%)		
	Confirmed mild case	3 (17.7%)	4 (23.5%)	10 (58.8%)		
	Confirmed severe case	1 (16.67%)	1 (16.67%)	4 (66.67%)		
Online courses (*N* = 1,298)	Not taking	178 (46.3%)	91 (23.7%)	115 (30.0%)	177.19	<0.001
	Taking school courses	45 (10.4%)	76 (17.5%)	313 (72.1%)		
	Taking company courses	108 (22.5%)	111 (23.1%)	261 (54.4%)		
Family environment	Cohesion	8.6 ± 0.9	8.3 ± 1.3	8.1 ± 1.4	26.75	<0.001
	Contradiction	1.6 ± 1.4	1.8 ± 1.5	2.2 ± 1.7	30.50	<0.001
Maternal emotions	SDS score	38.6 ± 0.1	38.8 ± 0.1	39.8 ± 0.1	4.21	0.040
	SAS score	33.5 ± 6.8	34.6 ± 7.1	35.3 ± 7.9	13.66	<0.001

*SDS, the Self-Rating Depression Scale; SAS, the Self-Rating Anxiety Scale; yrs, years; wks, weeks*.

*Analyzed by the Chi-square test or One-way ANOVA*.

**Figure 3:**
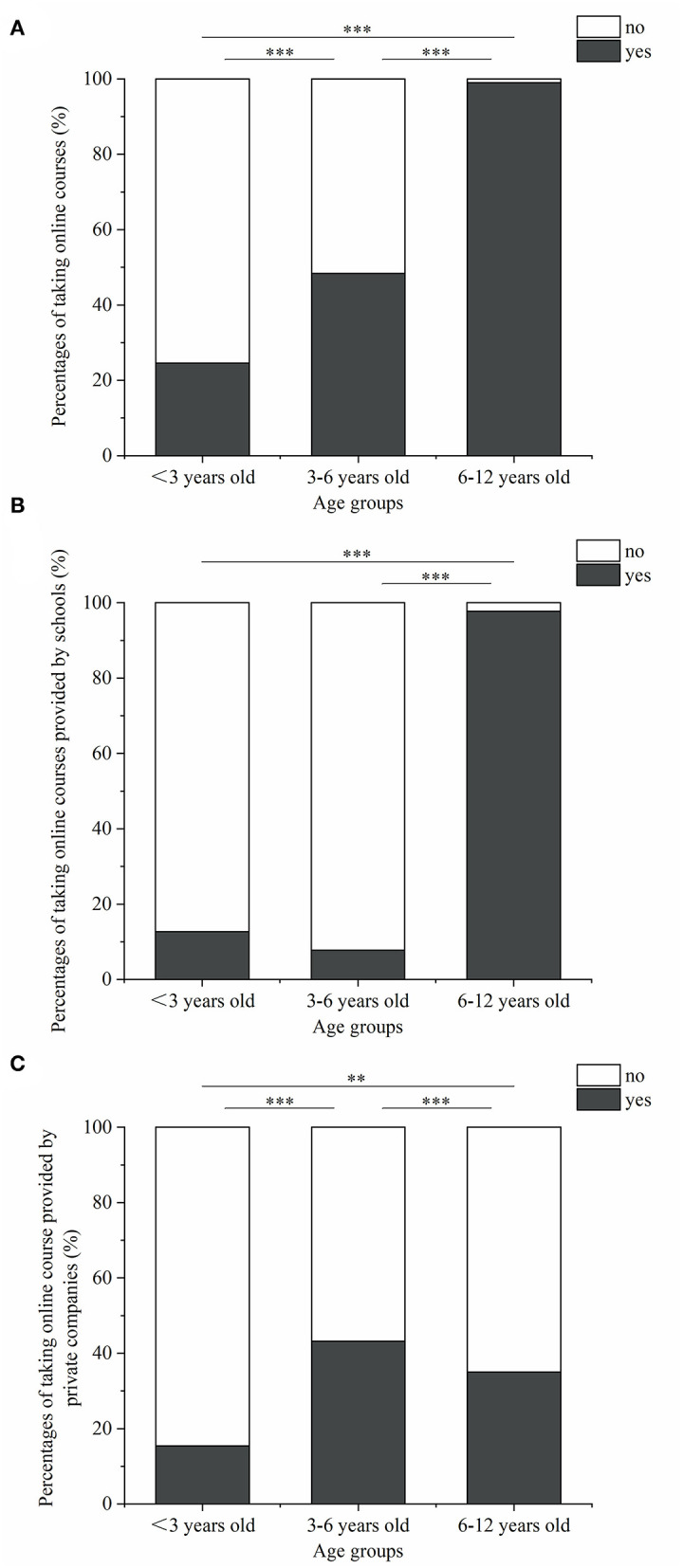
The current situations of child taking online courses. **(A)** Percentages of taking online courses; **(B)** percentages of taking online courses provided by school; **(C)** percentages of taking online courses provided by private companies. **P < 0.01; ***P < 0.001.

A total of 74.5% of the study children were involved in online courses. Children who took online courses had higher rates of excessive electronic use than children who didn't take. The percentages of taking online courses among children in nurseries, kindergartens and primary schools were 24.5, 48.4, and 99.0%, respectively (*P* < 0.001) (Figure 3). Online courses provided by children's own schools and by private education companies were 12.7 and 15.5% in nursery children, 7.8 and 43.2% in kindergarteners, and 97.7 and 35.0% in elementary-school students, respectively.

**Figure 4:**
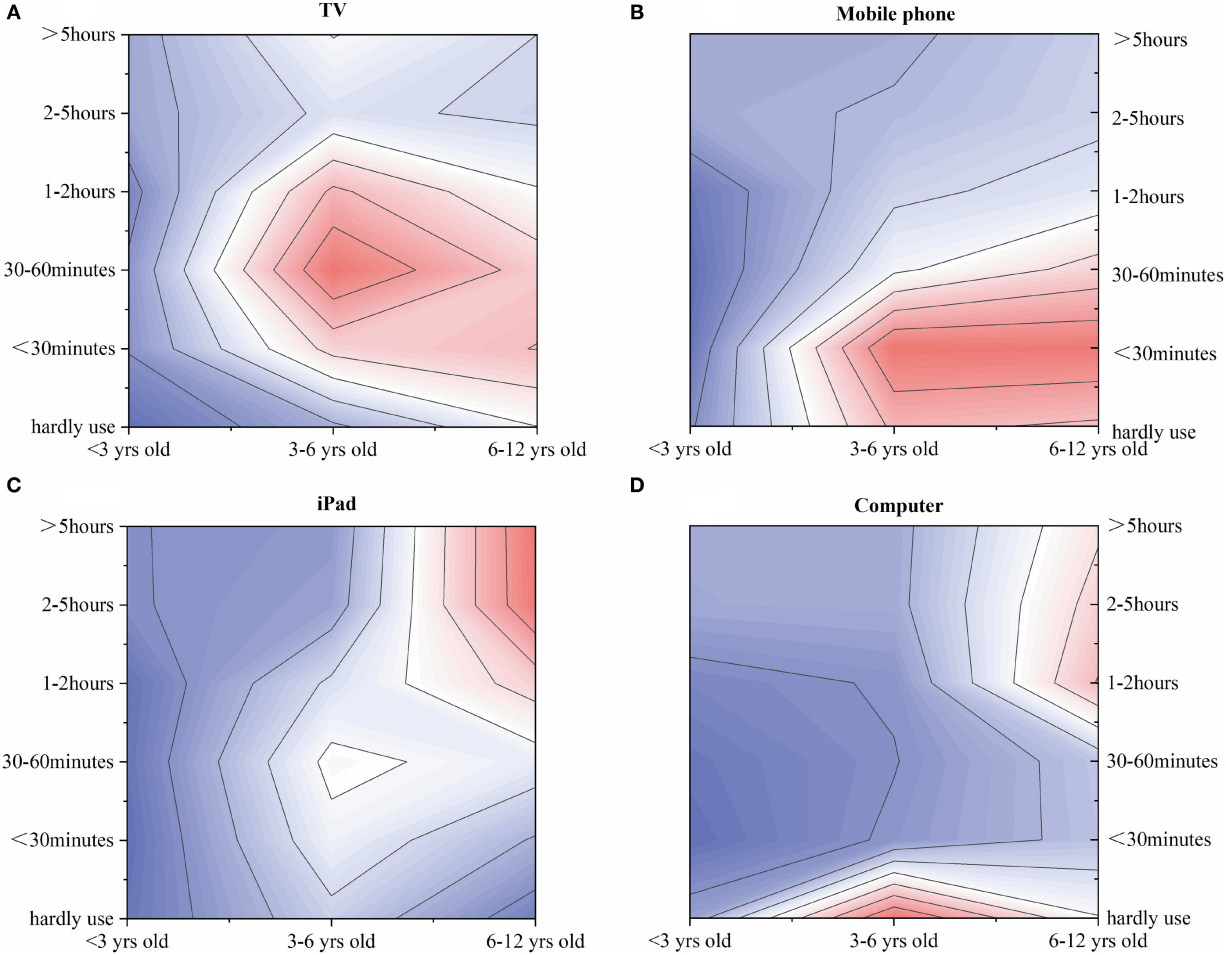
Use of different electronic devices among children of different ages. The red color indicated a higher distribution intensity, while the blue color indicated a lower distribution intensity, and the white color represented the transition or the distribution intensity between the red and blue colors. The distribution intensity decreased with the color getting lighter. **(A)** Child TV-watching per day. **(B)** Child mobile-phone usage per day. **(C)** Child iPad usage per day. **(D)** Child computer-usage per day.

The screen time of using the four kinds of electronic devices varied greatly among children of different ages (*P* < 0.001, Figure 4). Children in kindergartens spent more time watching TV than children of the other two age groups. Children in kindergartens and elementary schools spent similar amounts of time on mobile phones, but much longer times than children in nurseries. The rate of using iPad for over 2 h per day was the highest among elementary students (36.2%). The excessive computer uses mainly occurred in elementary-school students (17.9%). Adjusted associations between family environment and child excessive electronic use were described in Supplementary Figure 2. Both unadjusted and adjusted regression models showed that excessive use of portable internet devices was adversely correlated with family intimacy levels (*P* < 0.05). For primary-school children, the over-use of portable internet devices was positively associated with family conflict levels (*P* < 0.05).

### The Relationships Between Maternal Emotional Status and Child Over-Use of Electronic Devices

**Figure 5:**
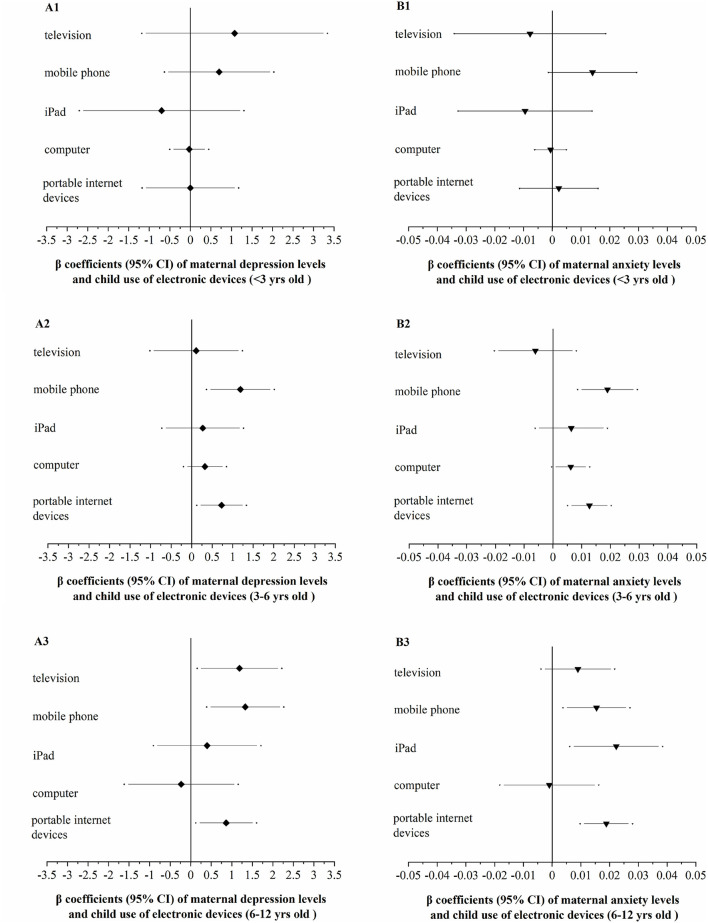
The adjusted relationships between maternal depression **(A)**/anxiety **(B)** and child over-use of electronic devices [β (95%CI)]. **(A/B-1)** For children <3 years old; **(A/B-2)** for children of 3–6 years old; **(A/B-3)** for children of 6–12 years old. Adjusted for child age, child gender, maternal age, maternal educational background, family structure, and home residency.

The relationships between electronic use and maternal emotional status among children of different age groups were shown in Figure 5. For kindergarteners, maternal depression and anxiety levels were significantly or marginally significantly associated with excessive use of mobile phones/portable internet devices. For children in elementary schools, maternal depression level was positively correlated with excessive use of TVs, mobile phones, and portable internet devices, and increased maternal anxiety level was related to excessive use of mobile phones and iPads, and portable internet devices. The adjusted associations between maternal depression/anxiety levels, family intimacy/conflict levels and child electronic over-use were provided in the Supplementary Tables 1, 2.

## Discussion

To cope with the ongoing COVID-19 pandemic, children and their mothers experienced instructions to stay at home and a considerable number of children took online courses. However, few studies focused on the association between maternal emotional status and child over-use of electronic devices during the COVID-19 pandemic. This study explored the characteristics of child over-use of electronic devices and the association between maternal emotional status and child over-use during the pandemic, which may help develop effective intervention strategies for reducing child screen time and improving mother-child interaction.

Compared with other studies conducted in China before or during the pandemic, this study reported a relatively high prevalence of depression (15.7%) among the study mothers. However, the prevalence of maternal anxiety (4.1%) was not higher than the prevalence reported during non-pandemic periods (31). For example, a large national survey on mental disorders among 32,552 respondents from 31 provinces across China between 2013 and 2015 reported that the prevalence of anxiety and major depression disorders were 7.6 and 3.4%, respectively (32). During the COVID-19 epidemic in China, Lei et al. found that 14.6% of survey respondents suffered from depression which was similar to the rate we reported in our study (15.7%) (33), while Peng et al. reported a lower prevalence of depression (6.21%) (34).

This study also reported a few potential risk factors for maternal depression and anxiety during the pandemic. Consistent with previous studies (35, 36), families with lower intimacy and higher conflict levels were associated with higher levels of maternal depression and anxiety. As a study specially targeting the COVID-19 pandemic, we found that lower levels of concern and awareness of healthy parenting during the outbreak, lower maternal education, and living in rural areas (vs. urban or suburban areas) were associated with increased levels of maternal depression and anxiety levels. Based on the knowledge-attitudes-practices model, our results suggested that lower levels of awareness (knowledge) and concern may translate into a lower likelihood of engaging in beneficial practices (37). Therefore, the provision of accurate and timely information about healthy parenting to mothers may be necessary for improving maternal emotional well-being during the pandemic. We also found that mothers with lower educational levels experienced greater psychological pressure, which was probably due to poor understanding of COVID-19 (such as transmission, causes, and symptoms) and unawareness of the related health-care information. These mothers may have faced more challenges when they were involved in the panic caused by the COVID-19 epidemic. In addition, living in rural areas was associated with less access to COVID-19 information compared with living in urban or suburban areas. Therefore, to alleviate maternal depression or anxiety levels, more accurate and timely information on COVID-19 available to mothers could be helpful, especially for mothers with lower education levels or living in rural areas.

This study demonstrated that over-use of electronic devices among young children was common, and the domestic-quarantine and online-teaching policies may have exacerbated the problem. In addition to online courses provided by schools, some parents asked children to take online courses developed by private educational companies to further improve their academic performances. This study showed that taking extra-curricular courses were particularly prevalent among preschoolers, reflecting parental worries about the possibility of a decline in child academic performance due to online education at home (32). This study found that older children, higher maternal educational levels, living closer to urban areas, and worse family environments were risk factors for child over-use of electronic devices. We speculate that older children usually take more online courses because of more study tasks, and have greater capability to use electronic devices, face more parent-child conflicts, and tend to release their feelings by spending more time on electronic devices. Inconsistent with previous studies showing that lower family socioeconomic status was a risk factor for child excessive use of electronic devices (38, 39), this study found that higher maternal education levels and urban residence (vs. rural or suburban areas) were positively associated with child over-use. These children may be better equipped to use electronic devices (the availability of the electronic devices at home, and the performances of Wi-Fi home networks). Consistent with previous studies showing exacerbated parent-child interaction was associated with excessive internet use, we also found that lower family intimacy levels (especially for younger children) and higher family conflict levels (especially for school-age children) were significantly associated with child over-use of electronic devices (40).

This study showed that maternal depression/anxiety was associated with a higher risk of child over-use of portable electronic devices. This association varied by child age. Previous studies have reported the association between maternal depression and child over-use of electronic devices during non-epidemic periods (38–41). For example, one study found a positive correlation between maternal depression and child binge television-watching (42). With regard to age differences in the associations of maternal emotions with child over-use, we found that maternal emotions had no significant associations with child over-use among children aged <3 years, whereas had important associations with excessive use of mobile phones among children in kindergartens and primary schools, and had important associations with iPad over-use in primary-school students. This age-dependent phenomenon may be explained by the fact that children aged 0–3 years have difficulties in using electronic devices independently. Thus, it may be difficult to observe such an association even if infants'/toddlers' behaviors were affected by maternal emotions. However, children in kindergartens and primary schools were usually independent users. Mother's emotion and psychological status may affect children in the following ways: On the one hand, depressed or anxious mothers may over-use or heavily rely on electronic devices themselves, setting an example for their children; these mothers may have inappropriate attitudes in caring for children or guiding children's behaviors, or use screen time as the compensation or substitution for insufficient maternal companion (electronic babysitter) (38, 39, 43, 44). On the other hand, children having depressed or anxious mothers may seek relief or self-entertainment from portable internet devices because portable devices were small and easy to carry and to go online with. The latter was supported by our findings in that higher maternal depression/anxiety levels were associated with worse family environments, which in turn were associated with higher rates of over-use of electronic devices among children.

This study also demonstrated that family environment may play a mediating role in the association of maternal emotional status (especially maternal depression levels) with child over-use of electronic devices. The family is the sum of relationships and behaviors among family members (41). Although no definite definition, family environment or functioning was usually regarded as the model/way how family members interacted, kept relationships, and solved problems (45). Consistent with our study showing an association between maternal emotional status and family environment, Xie et al. found that worse family environment (impaired family cohesion, higher levels of conflict and independence) was associated with worse maternal emotional status (46). In addition, consistent with our study showing an association between child electronic-device over-use and family environment, other study reported that poorer family-quality relationships were associated with increased severity of child problematic online gaming (47), and Huang et al. found that child behavioral problems may interact with their family environmental factors (28). Consistent with our study, Yang et al. reported that, as an important component of the family environment/functioning, parenting style may play a mediating role in the association of maternal work-family conflict and child problematic internet use (48).

This study had several advantages. To our knowledge, this study was among the first to focus on the association of maternal emotional status with child over-use of electronic devices during the COVID-19 pandemic. It addressed two major concerns (maternal emotional status, child over-use of electronic devices) in child health-care. We also explored the age differences in the association of maternal emotional status and child over-use of electronic devices, and suggested that the over-use of portable internet devices among young children deserved most attention. Second, up to now, a limited number of studies focused on child media exposure/screen time during the COVID-19 (49, 50), but only school-age children (6–12 years) were included in these studies. Because younger children may be more susceptible to the effects of over-use of electronic devices on behavioral or physical development (51, 52), and children under 6 years old also used the electronic devices (based on our study, 97.6% for the children of 3–6 years and 85.6% for the children under 3 years old), therefore, studies focusing on electronic-device use among children aged 0–6 years during the COVID-19 pandemic are important. In addition, we investigated and adjusted for a number of confounders in this study, but the magnitudes of most estimates did not change significantly with the adjustment, indicating that the results were not strongly confounded by these factors.

This study also had several limitations. First, the study schools were not selected at random, which may make the mother-child pairs not representative for the general population, and selection bias could not be ignored. Second, this was a cross-sectional study which made it impossible to elucidate the casual relationship between maternal emotional status and child over-use. Third, information of maternal emotional status and child electronic use was reported by the study mothers, which may be affected by maternal emotions and may cause recall bias (53). Fourth, although we tried to control for as many confounders as we can, there may still be some residual confounding (such as parental total screen hours) (54). Finally, although the sample size of this study met the minimum sample size requirement, longitudinal studies with larger sample sizes are still needed to further explore the association between maternal emotional status and child electronic-device over-use as well as the potential mediating role of the family environment.

## Conclusion

In the context of the quarantine and online education policies during the COVID-19 outbreak, among children aged 0–12 years, our study found that child over-use of electronic devices was common, especially among children aged ≥6 years, and the online-education may exacerbate the over-use. Significant associations between maternal anxiety/depression and over-use of portable internet devices (especially mobile phones) were observed, especially among preschoolers and school-aged students. The possibility of the involvement of family environment in the associations cannot be excluded. These findings may be helpful to develop recommendations or policies, to reduce child electronic over-use during the COVID-19 pandemic, and enhance children's physical and psychological health.

## Data Availability Statement

The raw data supporting the conclusions of this article will be made available by the authors, without undue reservation.

## Ethics Statement

The studies involving human participants were reviewed and approved by the Research Ethics Committee Board of the International Peace Maternity and Child Health Hospital. Written informed consent to participate in this study was provided by the participants' legal guardian/next of kin.

## Author Contributions

XG collected, analyzed the data, and drafted the results. YZ participated in organizing and drafting the manuscript. JX designed the study and revised the manuscript. YH provided support in data collection and offered guidance in this study. ZL reviewed and provided feedback for the manuscript. All authors read, provided feedback, and approved the final manuscript.

## Funding

All phases of this study were supported by the National Natural Science Foundation of China [81974486 and 81673189] (to JX), National Key Research and Development Project [2016YFC1000203] (to ZL), and Shanghai Jiao Tong University School of Medicine [20172016] (to JX).

## Conflict of Interest

The authors declare that the research was conducted in the absence of any commercial or financial relationships that could be construed as a potential conflict of interest.

## Publisher's Note

All claims expressed in this article are solely those of the authors and do not necessarily represent those of their affiliated organizations, or those of the publisher, the editors and the reviewers. Any product that may be evaluated in this article, or claim that may be made by its manufacturer, is not guaranteed or endorsed by the publisher.

## Supplementary Material

Supplementary Figure 1 The adjusted relationships between child over-use of electronic devices and family environment [β (95%CI)]. Adjusting for child age, child gender, maternal age, maternal educational background, family structure, and home residency.

Supplementary Figure 2 The adjusted relationships between family environment and maternal depression **(A)**/anxiety levels **(B)** [β (95%CI)]. Adjusting for child age, child gender, maternal age, maternal education, family structure, home residency.

Supplemental Table 1 The adjusted relationships between maternal emotional status and child over-use of electronic devices.

Supplemental Table 1.1 The adjusted relationships between maternal depression levels and child over-use of electronic devices. Adjusted for child age, child gender, maternal age, maternal educational background, family structure, and home residency. Analyzed by multi-variable logistic regression.

Supplemental Table 1.2 The adjusted relationships between maternal anxiety levels and child over-use of electronic devices. Adjusted for child age, child gender, maternal age, maternal educational background, family structure, and home residency. Analyzed by multi-variable logistic regression.

Supplemental Table 2 The adjusted relationships between family environment and child over-use of electronic devices.

Supplemental Table 2.1 The adjusted relationships between family intimacy and child over-use of electronic devices. Adjusted for child age, child gender, maternal age, maternal educational background, family structure, and home residency. Analyzed by multi-variable logistic regression.

Supplemental Table 2.2 The adjusted relationships between family contradiction and child over-use of electronic devices. Adjusted for child age, child gender, maternal age, maternal educational background, family structure, and home residency. Analyzed by multi-variable logistic regression.

## References

[1] Wu F Zhao S Yu B Chen YM Wang W Song ZG. A new coronavirus associated with human respiratory disease in China. Nature. (2020) 579:265–9. 10.1038/s41586-020-2008-3 32015508 PMC7094943

[2] Huang C Wang Y Li X Ren L Zhao J Hu Y. Clinical features of patients infected with 2019 novel coronavirus in Wuhan, China. Lancet. (2020) 395:497–506. 10.1016/S0140-6736(20)30183-5 31986264 PMC7159299

[3] CSSE. COVID-19 Dashboard by the Center for Systems Science and Engineering (CSSE) at Johns Hopkins University (JHU). (2021). Available online at: https://coronavirus.jhu.edu/map.html (accessed October 3, 2021).

[4] Wang F-S Zhang C. What to do next to control the 2019-nCoV epidemic? Lancet. (2020) 395:391–3. 10.1016/S0140-6736(20)30300-7 32035533 PMC7138017

[5] Wang G Zhang Y Zhao J Zhang J Jiang F. Mitigate the effects of home confinement on children during the COVID-19 outbreak. Lancet. (2020) 395:945–7. 10.1016/S0140-6736(20)30547-X 32145186 PMC7124694

[6] Brooks SK Webster RK Smith LE Woodland L Wessely S Greenberg N. The psychological impact of quarantine and how to reduce it: rapid review of the evidence. Lancet. (2020) 395:912–20. 10.1016/S0140-6736(20)30460-8 32112714 PMC7158942

[7] Wu Y Zhang C Liu H Duan C Li C Fan X. Perinatal depressive and anxiety symptoms of pregnant women along with COVID-19 outbreak in China. Am J Obstet Gynecol. (2020) 223:e1–240.e9. 10.1016/j.ajog.2020.05.009 32437665 PMC7211756

[8] Liu N Zhang F Wei C Jia Y Shang Z Sun L. Prevalence and predictors of PTSS during COVID-19 outbreak in China hardest-hit areas: Gender differences matter. Psychiatry Res. (2020) 287:112921. 10.1016/j.psychres.2020.112921 32240896 PMC7102622

[9] Lamb ME LC. Father–child relationships. In: Cabrera NJ Tamis-LeMonda CS, editors, Handbook of Father Involvement: Multidisciplinary Perspectives. New York, NY: Routledge; Shannon M. O. Wittig, Christina M. Rodriguez (2013). p. 119–34.

[10] Wittig S Rodriguez C. Emerging behavior problems: bidirectional relations between maternal and paternal parenting styles with infant temperament. Dev Psychol. (2019) 55:dev0000707. 10.1037/dev0000707 30742467 PMC6533133

[11] Collins C Landivar LC Ruppanner L Scarborough WJ. COVID-19 and the gender gap in work hours. Wiley Public Health Emerg Collection. (2020) 2020:12506. 10.1111/gwao.12506 32837019 PMC7361447

[12] Yang GY Huang LH Schmid KL Li CG Chen JY He GH. Associations between screen exposure in early life and myopia amongst chinese preschoolers. Int J Environ Res Public Health. (2020) 17:1056. 10.3390/ijerph17031056 32046062 PMC7037286

[13] Smahel D Wright MF Cernikova M. The impact of digital media on health: children's perspectives. Int J Public Health. (2015) 60:131–7. 10.1007/s00038-015-0649-z 25601331

[14] Sahu M Gandhi S Sharma MK. Mobile phone addiction among children and adolescents: a systematic review. J Addict Nurs. (2019) 30:261–8. 10.1097/JAN.0000000000000309 31800517

[15] Radesky J Christakis D. Media and young minds. Pediatrics. (2016) 138:e20162591. 10.1542/peds.2016-2591 27940793

[16] Swanson. Media use in school-aged children and adolescents. Pediatrics. (2016) 138:e20162592. 10.1542/peds.2016-2592 27940794

[17] Guo X Hua H Xu J Liu Z. Associations of childhood unintentional injuries with maternal emotional status during COVID-19. BMC Pediatr. (2021) 21:422. 10.1186/s12887-021-02846-2 34560850 PMC8460849

[18] Zung WW. A self-rating depression. Scale. Arch Gen Psychiatry. (1965) 12:63–70. 10.1001/archpsyc.1965.01720310065008 14221692

[19] Zung W. A rating instrument for anxiety disorders. Psychosomatics. (1971) 12:371–9. 10.1016/S0033-3182(71)71479-0 5172928

[20] Dunstan DA Scott N Todd AK. Screening for anxiety and depression: reassessing the utility of the Zung scales. BMC Psychiatry. (2017) 17:329. 10.1186/s12888-017-1489-6 28886698 PMC5591521

[21] Tao M Gao JF. Reliability and validity of the revised self-rating anxiety scale (SAS-CR). Chinese J Nerv Mental Dis. (1994) 5:301–3.

[22] Wu WY Self-rating anxiety scale. In: Zhang ZJ, editor, Behavioral Medicine Inventory Manual. (2005). p. 213–4.

[23] Zhang DX Luo JH Peng LZ Yu Z Li L Sun R. Factor analysis on survey results of the self-rating depression scale (SDS) in students. J Kunming Medical Univ. (2012) 5:61–3.

[24] Phillips MR. Family environment scale - chinese version (FES-CV). Chin Ment Health J. (1999). p. 134–42.

[25] Moos RH Insel PM Humphrey B. Preliminary Manual for Family Environment Scale, Work Environment Scale, Group Environment Scale. Palo Alto, CA: Consulting Psychologists Press (1974). 10.1037/t06503-000

[26] Wang XD Wang XL Ma H. Rating scales for mental health. Chin Ment Health J. (1999) 161–7.

[27] Ren Y Fang X Fang H Pang G Cai J Wang S. Predicting the adult clinical and academic outcomes in boys with ADHD: a 7- to 10-year follow-up study in China. Front Pediatr. (2021) 9:634633. 10.3389/fped.2021.634633 34408992 PMC8367416

[28] Huang Y Xu H Au W Xu C Wu K. Involvement of family environmental, behavioral, and social functional factors in children with attention-deficit/hyperactivity disorder. Psychol Res Behav Manag. (2018) 11:447–57. 10.2147/PRBM.S178080 30349411 PMC6183693

[29] Yu S Sussman S. Does smartphone addiction fall on a continuum of addictive behaviors? Int J Environ Res Public Health. (2020) 17:422. 10.3390/ijerph17020422 31936316 PMC7014405

[30] Fischer-Grote L Kothgassner OD Felnhofer A. Risk factors for problematic smartphone use in children and adolescents: a review of existing literature. Neuropsychiatr. (2019) 33:179–90. 10.1007/s40211-019-00319-8 31493233 PMC6901427

[31] WHO. Depression and Other Common Mental Disorders:Global Health Estimates. Geneva: World Health Organization (2017).

[32] Huang Y Wang Y Wang H Liu Z Yu X Yan J. Prevalence of mental disorders in China: a cross-sectional epidemiological study. Lancet Psychiatry. (2019) 6:211–24. 10.1016/S2215-0366(18)30511-X 30792114

[33] Lei L Huang X Zhang S Yang J Yang L Xu M. Comparison of prevalence and associated factors of anxiety and depression among people affected by versus people unaffected by quarantine during the COVID-19 epidemic in Southwestern China. Med Sci Monit. (2020) 26:e924609. 10.12659/MSM.924609 32335579 PMC7199435

[34] Peng M Mo B Liu Y Xu M Song X Liu L. Prevalence, risk factors and clinical correlates of depression in quarantined population during the COVID-19 outbreak. J Affective Disord. (2020) 275:119–24. 10.1016/j.jad.2020.06.035 32658813 PMC7330582

[35] Reid V Meadows-Oliver M. Postpartum depression in adolescent mothers: an integrative review of the literature. J Pediatr Health Care. (2007) 21:289–98. 10.1016/j.pedhc.2006.05.010 17825726

[36] Beck CT. Predictors of postpartum depression: an update. Nurs Res. (2001) 50:275–85. 10.1097/00006199-200109000-00004 11570712

[37] De Pretto L Acreman S Ashfold MJ Mohankumar SK Campos-Arceiz A. The link between knowledge, attitudes and practices in relation to atmospheric haze pollution in Peninsular Malaysia. PLoS ONE. (2015) 10:e0143655. 10.1371/journal.pone.0143655 26646896 PMC4672926

[38] Faltýnková A Blinka L Ševčíková A Husarova D. The Associations between family-related factors and excessive internet use in adolescents. Int J Environ Res Public Health. (2020) 17:51754. 10.3390/ijerph17051754 32182673 PMC7084393

[39] Wu CST Wong HT Yu KF Fok KW Yeung SM Lam CH. Parenting approaches, family functionality, and internet addiction among Hong Kong adolescents. BMC Pediatr. (2016) 16:130. 10.1186/s12887-016-0666-y 27538688 PMC4991114

[40] Li K Davison KK Jurkowski JM. Mental health and family functioning as correlates of a sedentary lifestyle among low-income women with young children. Women Health. (2012) 52:606–19. 10.1080/03630242.2012.705243 22860706 PMC3459328

[41] Duch H Fisher EM Ensari I Harrington A. Screen time use in children under 3 years old: a systematic review of correlates. Int J Behav Nutr Phys Act. (2013) 10:102. 10.1186/1479-5868-10-102 23967799 PMC3844496

[42] Park S Chang HY Park EJ Yoo H Jo W Kim SJ. Maternal depression and children's screen overuse. J Korean Med Sci. (2018) 33:e219. 10.3346/jkms.2018.33.e219 30127707 PMC6097071

[43] Chung L. Personal factors, internet characteristics, and environmental factors contributing to adolescent internet addiction: a public health perspective. Int J Environ Res Public Health. (2019) 16:4635. 10.3390/ijerph16234635 31766527 PMC6926822

[44] Bjelland M Soenens B Bere E Kovács É Lien N Maes L. Associations between parental rules, style of communication and children's screen time. BMC Public Health. (2015) 15:1002. 10.1186/s12889-015-2337-6 26428894 PMC4589944

[45] Zhang Y. Family functioning in the context of an adult family member with illness: a concept analysis. J Clin Nurs. (2018) 27:3205–24. 10.1111/jocn.14500 29700875 PMC6105391

[46] Xie M Wang X Zhang J Wang Y. Alteration in the psychologic status and family environment of pregnant women before and during the COVID-19 pandemic. Int J Gynaecol Obstet. (2021) 153:71–5. 10.1002/ijgo.13575 33403679 PMC9087655

[47] Schneider LA King DL Delfabbro PH. Family factors in adolescent problematic Internet gaming: a systematic review. J Behav Addict. (2017) 6:321–33. 10.1556/2006.6.2017.035 28762279 PMC5700711

[48] Yang HM Kim HR. Work-family conflict on children's internet addiction: role of parenting styles in Korean Working Mother. Int J Environ Res Public Health. (2021) 18:115774. 10.3390/ijerph18115774 34072195 PMC8199257

[49] Kim SJ Lee S Han H Jung J Yang SJ Shin Y. Parental mental health and children's behaviors and media usage during COVID-19-related school closures. J Korean Med Sci. (2021) 36:e184. 10.3346/jkms.2021.36.e184 34184439 PMC8239422

[50] Seguin D Kuenzel E Morton JB Duerden EG. School's out: parenting stress and screen time use in school-age children during the COVID-19 pandemic. J Affect Disord Rep. (2021) 6:100217. 10.1016/j.jadr.2021.100217 34514458 PMC8423665

[51] Beyens I Valkenburg PM Piotrowski JT. Screen media use and ADHD-related behaviors: Four decades of research. Proc Natl Acad Sci U S A. (2018) 115:9875–81. 10.1073/pnas.1611611114 30275318 PMC6176582

[52] Johnson JG Cohen P Smailes EM Kasen S Brook JS. Television viewing and aggressive behavior during adolescence and adulthood. Science. (2002) 295:2468–71. 10.1126/science.1062929 11923542

[53] Radesky JS Weeks HM Ball R Schaller A Yeo S Durnez J. Young Children's Use of Smartphones and Tablets. Pediatrics. (2020) 146:e20193518. 10.1542/peds.2019-3518 32482771 PMC7329252

[54] Sanders W Parent J Forehand R Sullivan AD Jones DJ. Parental perceptions of technology and technology-focused parenting: Associations with youth screen time. J Appl Dev Psychol. (2016) 44:28–38. 10.1016/j.appdev.2016.02.005 27795603 PMC5082753

