# Supplementary figures and images for: The Association of Maternal Emotional Status With Child Over-Use of Electronic Devices During the COVID-19 Pandemic
Source: Front Pediatr. 2021 Dec 6;9:760996. doi: 10.3389/fped.2021.760996 (PMC8685459; doi:10.3389/fped.2021.760996)

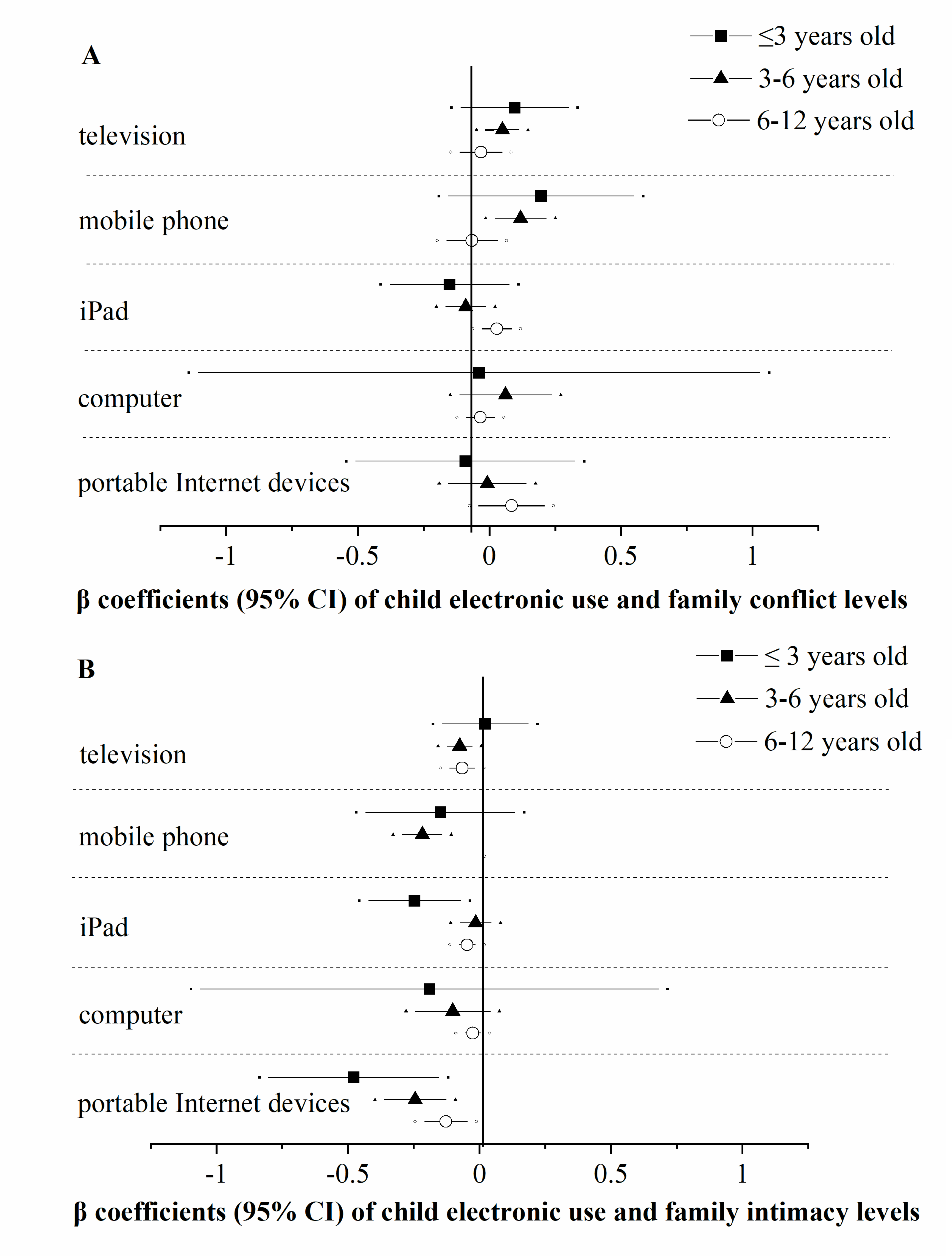

Supplement: Supplementary Figure 1 — The adjusted relationships between child over-use of electronic devices and family environment [β (95%CI)]. Adjusting for child age, child gender, maternal age, maternal educational background, family structure, and home residency. [file Image_1.TIF]

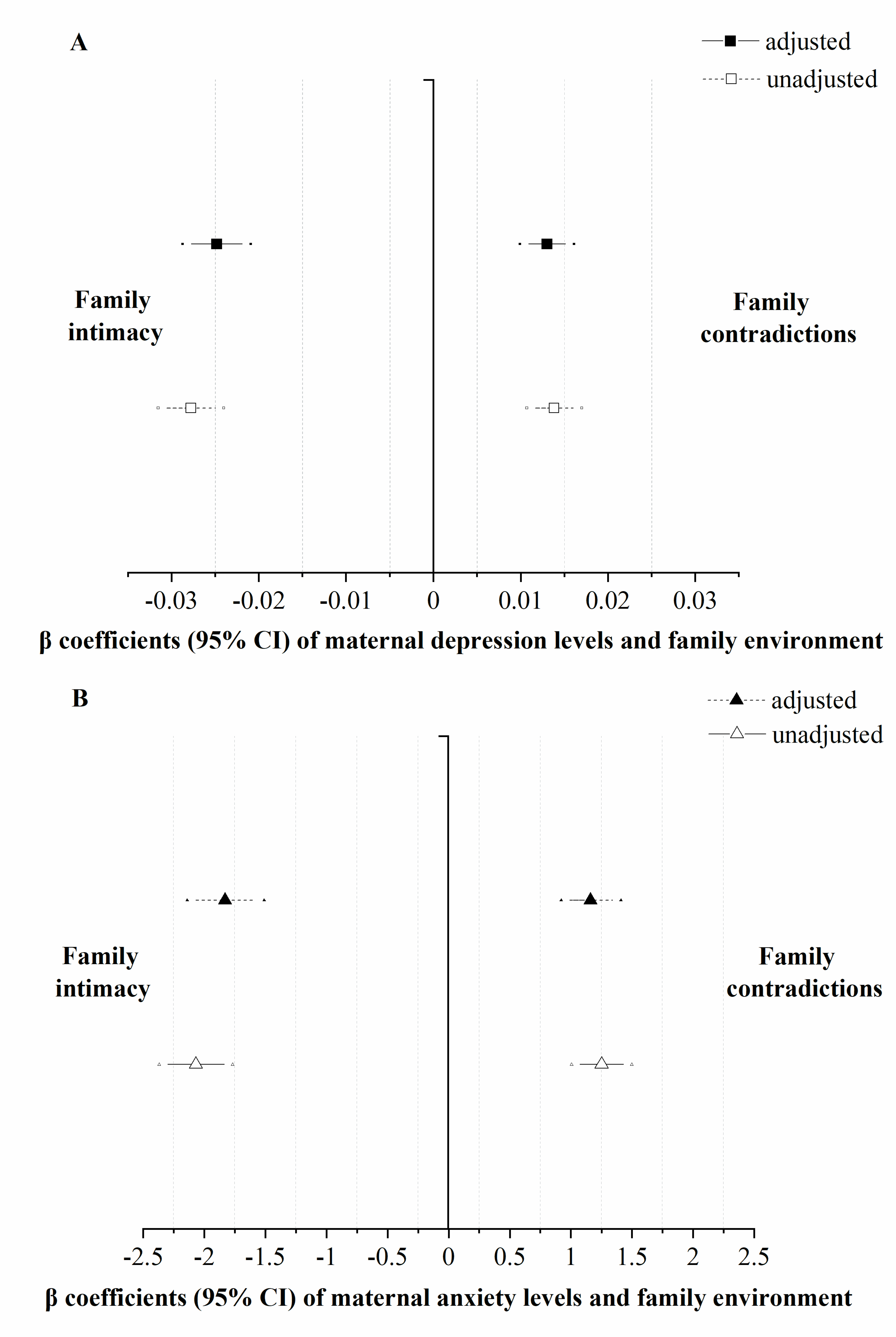

Supplement: Supplementary Figure 2 — The adjusted relationships between family environment and maternal depression (A)/anxiety levels (B) [β (95%CI)]. Adjusting for child age, child gender, maternal age, maternal education, family structure, home residency. [file Image_2.TIF]
