# Supplementary material for: The Association of Maternal Emotional Status With Child Over-Use of Electronic Devices During the COVID-19 Pandemic
Source: Front Pediatr. 2021 Dec 6;9:760996. doi: 10.3389/fped.2021.760996 (PMC8685459; doi:10.3389/fped.2021.760996)
Supplement: Supplemental Table 1 — The adjusted relationships between maternal emotional status and child over-use of electronic devices. [file Data_Sheet_1.docx]

**Supplemental Table 1. The adjusted relationships between maternal emotional status and child over-use of electronic devices.**

**Supplemental Table 1.1 The adjusted relationships between maternal depression levels and child over-use of electronic devices.**

|  |  | OR | β (95%CI) | | | *P* values |
| --- | --- | --- | --- | --- | --- | --- |
| Maternal depression levels |  | 7.323 | 1.991 (1.076, 2.907) | | | <0.001 |
| Child age |  | 1.474 | 0.388 (0.347, 0.429) | | | <0.001 |
| Child Gender | Male | 1.123 | 0.116 (-0.051, 0.283) | | | 0.173 |
|  | Female | REF |  |  |  |  |
| Maternal age |  | 0.988 | -0.012 (-0.032, 0.008) | | | 0.243 |
| Maternal education | ≤Junior High school | 0.533 | -0.63 (-1.105, -0.155) | | | 0.009 |
|  | High school | 0.738 | -0.304 (-0.661, 0.053) | | | 0.095 |
|  | College | 1.047 | 0.046 (-0.143, 0.234) | | | 0.633 |
|  | Post-graduate | REF |  |  |  |  |
| Family structure | Nuclear family | 1.143 | 0.134 (-1.433, 1.701) | | | 0.867 |
|  | Three generation family | 1.639 | 0.494 (-1.077, 2.065) | | | 0.538 |
|  | Separated parents | 1.791 | 0.583 (-1.240, 2.406) | | | 0.531 |
|  | Single parents | 2.111 | 0.747 (-0.980, 2.474) | | | 0.396 |
|  | Reconstituted family | REF |  |  |  |  |
| Residency | Urban | 0.935 | -0.067 (-0.793, 0.659) | | | 0.857 |
|  | Sub-urban | 0.909 | -0.095 (-0.819, 0.629) | | | 0.797 |
|  | Rural area | REF |  |  |  |  |

Adjusted for child age, child gender, maternal age, maternal educational background, family structure, and home residency.

Analyzed by multi-variable logistic regression.

**Supplemental Table 1.2 The adjusted relationships between maternal anxiety levels and child over-use of electronic devices.**

|  |  | OR | β (95%CI) | | | *P* values |
| --- | --- | --- | --- | --- | --- | --- |
| Maternal anxiety levels |  | 1.024 | 0.024 (0.012, 0.035) | | | <0.001 |
| Child age |  | 1.470 | 0.385 (0.343, 0.426) | | | <0.001 |
| Child Gender | Male | 1.123 | 0.116 (-0.051, 0.284) | | | 0.172 |
|  | Female | REF |  |  |  |  |
| Maternal age |  | 0.988 | -0.012 (-0.032, 0.008) | | | 0.243 |
| Maternal education | ≤Junior High school | 0.578 | -0.549 (-1.020, -0.078) | | | 0.022 |
|  | High school | 0.787 | -0.240 (-0.595, 0.115) | | | 0.186 |
|  | College | 1.062 | 0.060 (-0.128, 0.248) | | | 0.532 |
|  | Post-graduate | REF |  |  |  |  |
| Family structure | Nuclear family | 0.999 | -0.001 (-1.564, 1.561) | | | 0.999 |
|  | Three generation family | 1.430 | 0.358 (-1.208, 1.925) | | | 0.654 |
|  | Separated parents | 1.597 | 0.468 (-1.354, 2.289) | | | 0.615 |
|  | Single parents | 1.677 | 0.517 (-1.207, 2.242) | | | 0.557 |
|  | Reconstituted family | REF |  |  |  |  |
| Residency | Urban | 0.990 | -0.010 (-0.738, 0.718) | | | 0.979 |
|  | Sub-urban | 0.960 | -0.041 (-0.767, 0.684) | | | 0.911 |
|  | Rural area | REF |  |  |  |  |

Adjusted for child age, child gender, maternal age, maternal educational background, family structure, and home residency.

Analyzed by multi-variable logistic regression.

**Supplemental Table 2. The adjusted relationships between family environment and child over-use of electronic devices.**

**Supplemental Table 2.1 The adjusted relationships between family intimacy and child over-use of electronic devices.**

|  |  | OR | β (95%CI) | | | *P* values |
| --- | --- | --- | --- | --- | --- | --- |
| Family intimacy |  | 0.815 | -0.205 (-0.278, -0.132) | | | <0.001 |
| Child age |  | 1.470 | 0.385 (0.344, 0.426) | | | <0.001 |
| Child Gender | Male | 1.125 | 0.118 (-0.049, 0.285) | | | 0.167 |
|  | Female | REF |  |  |  |  |
| Maternal age |  | 0.986 | -0.014 (-0.034, 0.006) | | | 0.183 |
| Maternal education | ≤Junior High school | 0.551 | -0.596 (-1.070, -0.122) | | | 0.014 |
|  | High school | 0.739 | -0.303 (-0.659, 0.053) | | | 0.095 |
|  | College | 1.079 | 0.076 (-0.113, 0.264) | | | 0.432 |
|  | Post-graduate | REF |  |  |  |  |
| Family structure | Nuclear family | 0.923 | -0.080 (-1.645, 1.486) | | | 0.921 |
|  | Three generation family | 1.340 | 0.293 (-1.277, 1.863) | | | 0.715 |
|  | Separated parents | 1.209 | 0.190 (-1.632, 2.012) | | | 0.838 |
|  | Single parents | 1.536 | 0.429 (-1.302, 2.159) | | | 0.627 |
|  | Reconstituted family | REF |  |  |  |  |
| Residency | Urban | 1.034 | 0.033 (-0.693, 0.758) | | | 0.930 |
|  | Sub-urban | 0.943 | -0.059 (-0.782, 0.664) | | | 0.873 |
|  | Rural area | REF |  |  |  |  |

Adjusted for child age, child gender, maternal age, maternal educational background, family structure, and home residency.

Analyzed by multi-variable logistic regression.

**Supplemental Table 2.2 The adjusted relationships between family contradiction and child over-use of electronic devices.**

|  |  | OR | β (95%CI) | | | *P* values |
| --- | --- | --- | --- | --- | --- | --- |
| Family contradiction |  | 1.135 | 0.127 (0.070, 0.183) | | | <0.001 |
| Child age |  | 1.458 | 0.377 (0.336, 0.418) | | | <0.001 |
| Child Gender | Male | 1.138 | 0.129 (-0.038, 0.296) | | | 0.130 |
|  | Female | REF |  |  |  |  |
| Maternal age |  | 0.987 | -0.013 (-0.033, 0.007) | | | 0.198 |
| Maternal education | ≤Junior High school | 0.595 | -0.519 (-0.989, -0.048) | | | 0.031 |
|  | High school | 0.829 | -0.187 (-0.541, 0.168) | | | 0.302 |
|  | College | 1.092 | 0.088 (-0.100, 0.276) | | | 0.360 |
|  | Post-graduate | REF |  |  |  |  |
| Family structure | Nuclear family | 0.862 | -0.148 (-1.713, 1.418) | | | 0.853 |
|  | Three generation family | 1.242 | 0.217 (-1.352, 1.787) | | | 0.786 |
|  | Separated parents | 1.262 | 0.233 (-1.583, 2.048) | | | 0.802 |
|  | Single parents | 1.719 | 0.542 (-1.184, 2.269) | | | 0.538 |
|  | Reconstituted family | REF |  |  |  |  |
| Residency | Urban | 0.919 | -0.085 (-0.815, 0.645) | | | 0.820 |
|  | Sub-urban | 0.884 | -0.123 (-0.851, 0.604) | | | 0.740 |
|  | Rural area | REF |  |  |  |  |

Adjusted for child age, child gender, maternal age, maternal educational background, family structure, and home residency.

Analyzed by multi-variable logistic regression.
